# Recent Advances in Underlying Pathologies Provide Insight into Interleukin-8 Expression-Mediated Inflammation and Angiogenesis

**DOI:** 10.4061/2011/908468

**Published:** 2011-12-22

**Authors:** Basit Saleem Qazi, Kai Tang, Asma Qazi

**Affiliations:** ^1^Department of Orthopedic Surgery Spine Unit, First Affiliated Hospital of Dalian Medical University, 222 Zhongshan Road, Dalian, Liaoning 116011, China; ^2^Department of Pathology and Pathophysiology, Dalian Medical University, 9 West Section Lvshun South Road, Lvshun District, Dalian, Liaoning 116044, China

## Abstract

Interleukin-8 has long been recognized to have anti-inflammatory activity, which has been established in various models of infection, inflammation, and cancer. Several cell types express the receptor for the cytokine IL-8 and upon its recognition produce molecules that are active both locally and systemically. Many different types of cells, in particular monocytes, neutrophils, epithelial, fibroblast, endothelial, mesothelial, and tumor cells, secrete IL-8. Increased expression of IL-8 and/or its receptors has been characterized in many chronic inflammatory conditions, including psoriasis, ARDS, COPD, and RA as well as many cancers, and its upregulation often correlates with disease activity. IL-8 constitutes the CXC class of chemokines, a potent chemoattractant and activator of neutrophils and other immune cells. It is a proangiogenic cytokine that is overexpressed in many human cancers. Therefore, inhibiting the effects of IL-8 signaling may be a significant therapeutic intervention.

## 1. Introduction

IL-8 is secreted by multiple cell types, including monocytes, neutrophils, epithelial, fibroblast, endothelial, mesothelial, and tumor cells. It is released from several cell types in response to an inflammatory stimulus [1]. IL-8 plays an important role in inflammation and wound healing [2] and has a capacity to recruit T cells as well as nonspecific inflammatory cells into sites of inflammation by activating neutrophils [3]. It also stimulates *α*-smooth muscle actin production in human fibroblasts [4]. Furthermore, IL-8 is chemotactic for fibroblasts, accelerates their migration, and stimulates deposition of tenascin, fibronectin, and collagen I during wound healing *in vivo* [4]. This paper summarizes current knowledge on the central role of IL-8 in different pathologies. The experimental results and questions posted in research work on IL-8 are covered here, and the potential roles of IL-8 as part of a complex cytokine network in wound healing, angiogenesis, and several cancers are discussed here.

## 2. Expression of IL-8 in Immune System

In many cell types, the synthesis of IL-8 is strongly stimulated by IL-1 and TNF-*α*. In human skin fibroblasts, the expression of IL-8 is enhanced by leukoregulin. The synthesis of IL-8 is induced also by phytohemagglutinins, concanavalin A, double-stranded RNA, phorbol esters, sodium urate crystals, viruses, and bacterial lipopolysaccharides. The expression of IL-8 from resting and stimulated human blood monocytes is upregulated by IL-7 [5].

In chondrocytes, the synthesis of IL-8 is stimulated by IL1-*β*, TNF-*α*, and bacterial lipopolysaccharides. In human astrocytes, the synthesis and secretion of IL-8 is induced by IL-1 and TNF-*α*. Glucocorticoids, IL-4, TGF-*β*, inhibitors of 5′ lipoxygenase, and 1.25(OH)2 vitamin D3 inhibit the synthesis of IL-8. IL-8 is constitutively and commonly produced by various carcinoma cell lines, and this synthesis may be related to the elevation of serum IL-8 in patients with hepatocellular carcinoma. In epithelial, endothelial, and fibroblastic cells, secretion of IL-8 is induced by IL-17 [6]. 

## 3. Protein Characteristics

IL-8 is an 8.4 kDa nonglycosylated protein produced by processing of a precursor protein of 99 amino acids belonging to the CXC subfamily of chemokines which is characterised by two essential cysteine residues, separated by a third intervening amino acid [7, 8]. There are two major forms of IL-8, that are the 72-amino acid monocyte-derived form, predominant in cultures of monocytes and macrophages, and the endothelial form which has five extra N-terminal amino acids, predominating in cultures of tissue cells such as endothelial cells and fibroblasts [9, 10].

Longer forms of IL-8 (79 and 77 amino acids) and shorter forms (69 amino acids) have been isolated also from conditioned medium of lymphocytes stimulated with bacterial lipopolysaccharides, fibroblasts stimulated by IL-1 or TNF, and polyI: C-stimulated endothelial cells. The predominant form of IL-8 produced by endothelial cells (and also by anchorage-dependent cells and human glioblastoma cells) is the 77-amino acid variant. IL-8 (6–77) has a 5–10-fold higher activity on neutrophil activation, IL-8 (5–77) has increased activity on neutrophil activation, and IL-8 (7–77) has a higher affinity to receptors CXCR1 and CXCR2 as compared to IL-8 (1–77), respectively [11].

## 4. IL-8 Structure

The human IL-8 gene (SCYB8) has a length of 5.1 kb and maps to human chromosome 4q12-q21. The mRNA consists of a 101-base 5′ untranslated region, an open reading frame of 297 bases and a long 3′ untranslated region of 1.2 kb. The 5′ flanking region of the IL-8 gene contains potential binding sites for several nuclear factors including activation factor-1, activation factor-2, IFN regulatory factor-1, hepatocyte nuclear factor-1, a glucocorticoid-responsive element, and a heat shock element [12, 13].

## 5. IL-8 and Receptors

IL-8 receptors are a member of a G-protein-coupled receptor protein family. There are at least two different IL-8 receptor types. The type 1 receptor specifically binds IL-8 (Kd = 0.8–4 nM). The type 2 receptor (Kd for IL-8 = 0.3–2 nM) also binds the IL-8-related factors like MGSA (melanoma growth stimulatory activity), GRO, MIP-2 (macrophage inflammatory protein), and NAP-2 (neutrophil-activating protein-2). Both receptor genes map to human chromosome 2q35 [13–15]. 

## 6. Biological Functions and Expression of IL-8

The activities of IL-8 are not species specific. Human IL-8 is also active in animal cells. The biological activities of IL-8 resemble those of a related protein, NAP-2 (neutrophil-activating protein-2). It differs from all other cytokines in its ability to specifically activate neutrophil granulocytes where it causes a transient increase in cytosolic calcium levels and the release of enzymes from granules. IL-8 also enhances the metabolism of ROS (reactive oxygen species) and increases chemotaxis and the enhanced expression of adhesion molecules [16].

IL-8 alone does not release histamines. It actually inhibits histamine release from human basophils induced by histamine-releasing factors, CTAP-3 (connective tissue activating protein-3), and IL-3 [17]. IL-8 is involved also in pain meditation [18]. The intravenous administration of IL-8 in baboons causes a severe granulocytopenia followed by granulocytosis which persists as long as sufficient IL-8 levels are maintained [19].

IL-8 is chemotactic for all known types of migratory immune cells. IL-8 inhibits the adhesion of leukocytes to activated endothelial cells and therefore possesses anti-inflammatory activities. The 72-amino acid form of IL-8 is approximately tenfold more potent in inhibiting adhesion of neutrophils than the 77-amino acid variant [20].

IL-8 is a mitogen for epidermal cells, and *in vivo* it strongly binds to erythrocytes. This absorption may be of physiological importance in the regulation of inflammatory reactions since IL-8 bound to erythrocytes no longer activates neutrophils. Macrophage-derived IL-8 supports angiogenesis and plays role in disorders such as rheumatoid arthritis, tumor growth, and wound healing that critically depend on angiogenesis [21].

Simonet et al. (1994) have studied transgenic mice overexpressing IL-8. Elevated serum IL-8 levels were found to correlate with increases in circulating neutrophils and decreases in L-selectin expression on the surface of blood neutrophils. The accumulation of neutrophils was observed in the microcirculation of the lung, liver, and spleen. Neutrophil extravasation, plasma exudation, or tissue damage was absent [22].

IL-8 has been implicated in a number of inflammatory diseases, such as CF [23], ARDS (adult respiratory distress syndrome) [24], COPD (chronic obstructive pulmonary disease), and asthma [25]. The airway epithelium is one of several sources of IL-8 in the airway, and it serves as a barrier against invading microorganisms. Airway epithelial release of IL-8 contributes to host defense by promoting neutrophil chemotaxis and airway inflammation [26].

## 7. Clinical Significance

Inflammation is the single greatest cause of pain. The first inflammatory mediators recognized to have potent hyperalgesic properties was bradykinin [27], since then a host of inflammatory medicators have been identified which can produce hyperalgesia, including prostaglandins, leukotrienes, serotonin, adenosine, histamine, IL-1, IL-8, and NGF (nerve growth factor). 

Cytokines are produced by leukocytes in response to exposure to bacterial toxins or to inflammatory medicators [28]. IL-8 has also been found to produce a sympathetic-dependent hyperalgesia which does not appear to be medicated by prostaglandin [18, 29].

IL-8 was shown to be angiogenic factor in 1992 [21, 30]. Kitadai et al. Found high levels of IL-8 in six of eight carcinoma cells and lines and 32 of 39 gastric carcinoma specimens as compared to normal mucosal control. The levels of IL-8 correlated strongly with the specimen vascularity [31]. IL-8 was shown to be major inducer of neovascularisation of squamous cell carcinoma by lingen et al. [32]. IL-8 also plays a significant role in other cancer by mediating angiogenesis and tumorigenesis. IL-8 is produced by a wide panel of human cancer cells including colon [10], melanoma [33], prostate [34], ovary [35, 36], or breast [37–40]. 

### 7.1. IL-8 and Inflammatory Diseases

#### 7.1.1. Proinflammatory Effects of IL-8

IL-8 is an oxidative stress-responsive proinflammatory chemokine, released from epithelial cells following particle-induced oxidative stress leading to neutrophil influx and inflammation [41, 42]. IL-8 is a potent chemoattractant and activator of neutrophils, the transcription of which is NF-*κ*B dependent [43].

Proinflammatory stimuli are considered to be a major regulator of IL-8 levels in response to injury. IL-8 is involved in many of the wound healing processes. It not only serves as a chemotactic factor for leukocytes and fibroblasts but also stimulates fibroblast differentiation into myofibroblasts and promotes angiogenesis [44, 45].

IL-8 is a proinflammatory cytokine that is upregulated by different cellular stress stimuli [46]. Human cells are characterized by their marked capacity for varying the expression levels of IL-8, allowing modulating the concentration of this cytokine to control the degree of neutrophil infiltration in the injured tissue [46]. The expression of IL-8 is regulated at both transcriptional and posttranscriptional levels [47], and the main MAPK pathways (p38, MEK1/2, and JNK) play a significant role in the release of IL-8 during the inflammatory process [46].

#### 7.1.2. Enhancement of Corneal Wound Healing

The induction of IL-8 facilitates an early innate immune response to infection in the corneal stroma and represents an elementary defense mechanism in corneal wound healing [48]. It enhances healing by rapidly chemoattracting leukocytes and fibroblasts into the wound site, stimulating the latter to differentiate into myofibroblasts. In turn, myofibroblasts are critical for wound contraction and closure and for the production of extracellular matrix molecules, which leads to development of granulation tissue [44].

The role of PDGF in corneal wound healing [49] and IL-8-mediated neutrophil chemotaxis [50] has been previously documented. It enhances healing by rapidly chemoattracting leukocytes and fibroblasts into the wound site [2], where PDGF increases IL-8 chemokine secretion twofold in human corneal fibroblasts, indicating that IL-8 is involved in PDGF-mediated corneal wound healing. Both human corneal keratocytes and epithelial cells have been shown to synthesize and release IL-8 following cytokine stimulation and/or infection [51].

#### 7.1.3. Proliferation in Arthritis

IL-8 may be of clinical relevance in psoriasis and rheumatoid arthritis. Elevated concentrations are observed in psoriatic scales, and this may explain the high proliferation rate observed in these cells. IL-8 may be also a marker of different inflammatory processes [52]. 

IL-8 (and also IL-1 and IL-6) probably plays a role in the pathogenesis of chronic polyarthritis since excessive amounts of this factor are found in synovial fluids [53]. The activation of neutrophils may enhance the migration of cells into the capillaries of the joints. These cells are thought to pass through the capillaries and enter the surrounding tissues thus causing a constant stream of inflammatory cells through the joints [54].

#### 7.1.4. Role in Myelodysplastic Syndrome

Human recombinant IL-8 has shown that the lesion responsible for defective functions of neutrophils in patients with myelodysplastic syndrome can be restored without stimulating myeloid progenitor cells. IL-8 may be able, therefore, to reduce the risks of lethal infections in these patients without the potential risk of stimulating leukemic clones [55].

#### 7.1.5. Gastric Mucosal Injury and Cancer Progress

In the human gastric mucosa, elevated levels of ROS are associated with Helicobacter pylori infection [56], and it leads to oxidative DNA damage in the gastric mucosa thereby contributing to mucosal injury and promoting carcinogenesis [57, 58]. H. pylori infection is also associated with increased gastric mucosal cytokine expression including IL-8 [59, 60] and TNF-*α* [61, 62].

TNF-*α* is an endogenous mediator of proinflammatory cytokine stimulation and can induce ROS [63] and stimulate the induction of various genes involved in inflammation [64] including IL-8. IL-8 is an important mediator of H. pylori-associated neutrophil infiltration and gastric inflammation [57]. ROS modulates IL-8 secretion in gastric epithelial cells, suggesting that IL-8 gene expression in the gastric mucosa is redox sensitive [65].

Although a regulatory role of TNF-*α* in epithelial cell repair has been described [66], it is well established that TNF-*α* stimulates IL-8 and contributes to epithelial cell injury and apoptosis [63].

#### 7.1.6. Increased BAL Fluid in ALI and COPD

In human ALI (acute lung injury), neutrophil infiltration is an early and important pathophysiological event, and IL-8 appears to have an important role in mediating this process [67, 68]. Clinical research demonstrated increased IL-8 levels in serum and BAL (bronchoalveolar lavage) fluid of patients with ALI [69, 70]. Increased BAL fluid levels of IL-8 predicted the development of ALI in at-risk patient populations and are associated with increased mortality in patients with ALI [71]. In animal models of ALI, administration of IL-8 antibody conferred protection [72]. IL-8 is also produced by respiratory epithelium [26]. 

Studies involving alveolar macrophages, U937 cells, isolated peripheral blood monocytes, and human whole blood demonstrated that hyperoxia modulates IL-8 gene expression [73]. Oxidant stress other than hyperoxia was previously described to induce IL-8 expression in respiratory epithelial cells. DeForge et al. [74] and Lakshminarayanan et al. [75] hyperoxia alone had a minimal effect on IL-8 gene expression. However, combination of hyperoxia and TNF-a synergistically increased IL-8 gene expression.

COPD (*chronic obstructive pulmonary disease*) cigarette smoke can also induce airway inflammation. It has been shown to activate proinflammatory transcription factors NF-*κ*B and activator protein (AP)-1 [76] as well as to upregulate the expression of TNF-*α* and IL-8, proinflammatory mediators associated with COPD [77].

#### 7.1.7. Prevention of Lung Epithelial Cells Injury

TNF-*α* levels are markedly elevated in BAL fluid from patients with ARDS [78], and TNF-*α* levels are associated with increased IL-8 levels. TNF-*α* is a major inducer of IL-8 expression in lung epithelial cells [26]. Neutralizing IL-8 antibodies prevented lung injury in animal models of lung disease, indicating IL-8 is an important mediator of lung injury [79, 80]. IL-8 gene expression is induced by a wide variety of agents including cytokines, growth factors, bacterial and viral products, oxidants, and others [81]. Induction of IL-8 gene expression is subject to both transcriptional and posttranscriptional regulation in a cell/tissue-and stimulus-specific manner [46, 81].

In lung epithelial cells, TNF-*α* activates IL-8 promoter activity via recruitment of NF-*κ*B to a TNF-*α* response element consistent with a role for transcriptional mechanisms in the induction of IL-8 gene expression in lung epithelial cells [82].

#### 7.1.8. Signalling in Cystic Fibrosis

IL-8 drives the inflammatory response in cystic fibrosis (CF) which is an autosomal recessive disorder caused by mutations in the gene encoding the cystic fibrosis transmembrane conductance regulator (CFTR) [83].

BAL fluid in patients with CF contains increased levels of proinflammatory cytokines and neutrophils. IL-8 levels attribute to activate NF-*κ*B [84]. Prostaglandin E2 (PGE-2) is a potent mediator of inflammation produced by cyclooxgenation of arachidonic acid, and its hypersecretion results in elevated IL-8 secretion through unidentified signaling pathway [85].

In human T lymphocytes, PGE-2 induces C/EBP homologous protein (CHOP) transcription factor that binds to the IL-8 promoter [85]. CHOP is a growth arrest and DNA damage-inducible gene 153 (GADD153) protein. PGE-2 mediates the IL-8 inflammatory response in CF cells through the CHOP transcription factor. The inflammatory response in CF contributes to neutrophil-driven lung destruction [86, 87]. Several cytokines, such as IL-1b, TNF-**α**, IFN-**δ**, and bacterial products, induce IL-8 release from airway epithelial cells [88], thus exacerbating the baseline inflammatory milieu in CF.

Much of the PGE-2 in airways is likely to be derived from the epithelium [89], and the stimulation of chloride secretion in airway epithelial cells by proinflammatory mediators such as bradykinin (BK) occurs through the induced release of PGE-2 (26). Moreover, BK induces IL-8 secretion in non-CF and CF human airway epithelia [90].

The physiologic as well as pathologic concentrations up to 100 mM PGE-2 upregulate endogenous IL-8 expression in human intestinal epithelial cells [91] and enhance IL-8 production in human synovial fibroblasts stimulated with IL-1b [92].

#### 7.1.9. Increased Expression in Asthma

IL-8 plays an important role in inflammatory lung diseases like bronchial asthma or severe infections caused by respiratory syncytial virus (RSV), and during infancy it might lead to the development of recurrent wheezing and/or bronchial asthma [93]. Increased concentrations of IL-8 are found in the BAL fluid and sputum of asthmatic patients [13]. In addition, repeated administration of IL-8 into the airways induces bronchial hyperreactivity in guinea pigs [94]. Genetic association of IL-8 has been described with asthma [95] and RSV bronchiolitis [96].

IL-8 binds with high affinity to two different receptors: IL-8 receptor *α* (IL-8RA, CXCR1) and *β* (IL-8RB, CXCR2). These closely related proteins are members of the super family of receptors, which couple to guanine nucleotide-binding proteins. IL-8RA is localized on chromosome 2q35 [13], where linkage to total serum IgE levels in asthmatics has been described [94]. Association of IL-8RA polymorphisms has recently been described with asthma and chronic obstructive pulmonary disease [95]. However, IL-8RA polymorphisms do not play a major role, neither in the development of severe RSV infections nor in asthma.

#### 7.1.10. Increased Expression in the Colon Mucosa with Inflammatory Bowel Disease

IL-8 is produced in the colonic lamina propria of patients with inflammatory bowel disease.

There is no difference in IL-8 protein concentrations between inflamed mucosa of patients with Crohn's disease or ulcerative colitis. IL-8 does thus not permit the differentiation between these two diseases entities. Mucosal IL-8 protein and IL-8 mRNA concentrations are correlated with the degree of inflammation. IL-8 mRNA is strongly expressed by intestinal inflammatory cells but not by intestinal epithelial cells suggesting that virtually all IL-8 is produced by interstitial inflammatory cells [96].

An imbalance of the intestinal immune system with a shift towards proinflammatory mediators is a characteristic feature of inflammatory bowel diseases [97]. Among the proinflammatory cytokines, IL-8 together with IL-l and tumour necrosis factor play an important part. 

IL-8 is synthesised by various colonic cancer cell lines like HT-29 cells or Caco-2 cells [98]. Evidence has also been provided that isolated normal intestinal epithelial cells may synthesise IL-8 [98].

An increased synthesis of IL-8 has been described in the mucosa of patients with inflammatory bowel disease. Where Mahida and coworkers [99] found enhanced mucosal tissue concentrations of IL-8 essentially only in patients with ulcerative colitis but not in patients with Crohn's disease, Izzo et al. [100] detected increased concentrations of IL-8 also in the colonic mucosa from patients with Crohn's disease.

#### 7.1.11. Promotion of Endometriosis Pathogenesis

IL-8 is representative of *α*-chemokine group and is a chemotactic and angiogenic factor [101]. It acts as an endometrial autocrine and paracrine factor and regulates many physiologic processes such as menstruation and remodeling of endometrium [102]. In addition, IL-8 also contributes to the pathogenesis of endometriosis by promoting a vicious cycle of endometrial cell attachment, invasion, immune protection, cell growth, and further secretion [103].

The presence of inflammation and neovascularization observed in and around ectopic endometrial implants and the presence of inflammatory neutrophils in these lesions [104] is compatible with the biological actions of IL-8 [105]. IL-8 is detectable in the peritoneal fluid of most women with an active ovarian cycle, and it is a normal constituent of peritoneal fluid in women with and without endometriosis. The concentration of IL-8 in the peritoneal fluid was higher in women with endometriosis compared to women without, and that difference was statistically significant as has been reported previously [106, 107].

Peripheral blood macrophages from endometriosis patients produced increased concentrations of IL-8 [101]. In women with early endometriosis (American Fertility Society Stage 1), IL-8 concentrations is much high as compared to women with later stages of the disease. It can be speculated that this may implicate IL-8 in the induction of the disease, and it is conceivable that other chemokines participate in the chronic phase of endometriosis. There are a number of potential cellular sources of IL-8 in endometriosis. Enhanced production by peritoneal macrophages has been found [107], but normal endometrial gland cells [108] and stromal cells [109] also produce IL-8 that can be enhanced by proinflammatory mediators. In normal nonpregnant endometrium, IL-8 was found to be localized perivascularly [102], suggestive of a direct role upon endothelial cells as well as a function in presenting a fixed chemotactic stimulus to circulating leukocytes.

#### 7.1.12. Intervertebral Disc Causing Low Back Pain

Human NP (nucleus pulposus) produces IL-8. Significant quantities of IL-6, IL-8, and PGE2 were produced by both the sciatica and low back pain groups.

Burke et al. studied the production of inflammatory mediators in disc tissues in a similar group of patients [110] and compared the levels of IL-6, IL-8, and PGE2 in their disc tissue from patients undergoing discectomy for sciatica with those from patients undergoing fusion for discogenic low back pain which showed that more IL-6, IL-8, and PGE2 are produced by discs from patients with low back pain compared with discs from patients with sciatica. There was a trend towards less exposure of the NP in the group with low back pain only compared with those with sciatica introducing a bias towards higher levels of mediator production in the latter [111].

The rates of production of IL-6 and IL-8 in the AI and EXT categories of discs in low back pain are much higher than those found in those with sciatica. A combination of the innervation of the NP and increased production of proinflammatory mediators suggests that the mechanism for discogenic low back pain may be the induction of hyperalgesia in the newly innervated degenerating NP. Both IL-8 and PGE2 are known to induce hyperalgesia [112].

### 7.2. IL-8 and Cancer

The extensive effects of increased IL-8 activity on tumor pathogenesis make it a unique therapeutic target in cancer therapy. For example, IL-8 promotes tumor growth, angiogenesis, and metastasis in murine models of several cancers [113]. Moreover, blocking IL-8 activity with a monoclonal antibody has been shown to decrease tumor growth in two murine cancer models [114]. Blockade of IL-8 expression in some human melanoma cell lines by antisense RNA has shown that IL-8 functions as an autocrine growth modulator for these cells [33].

#### 7.2.1. High Expression in Ovarian Cancer

IL-8 is expressed at high levels in ovarian cancer cells where expression is correlated with tumorigenicity [115]. IL-8 is overexpressed in most human cancers, including ovarian carcinoma [116]. Induction of IL-8 expression is mediated primarily by the transcription factor NF-*κ*B [117]; however, the Src/signal transducer and activator of transcription 3 (Stat3) pathways may also promote IL-8 production independent of NF-*κ*B [118]. High tumor IL-8 expression is significant in ovarian cancer associated with advanced tumor stage and high-tumor grade. The higher the IL-8, the poorer the survival rate. IL-8 overexpression in ovarian cancer is associated with decreased patient survival and is an independent prognostic factor for poor clinical outcome that targeted therapy with IL-8 siRNA-DOPC in combination with chemotherapy effectively reduced tumor growth in both chemotherapy-sensitive and chemotherapy-resistant ovarian cancer models [119]. these antitumor effects are likely due to a reduction in proangiogenic factors present in the tumor microenvironment that led to decreased angiogenesis and tumor cell proliferation following silencing IL-8 expression. IL-8 may be a potential therapeutic target in ovarian cancer. IL-8 overexpression is reported in multiple malignancies and is frequently associated with poor clinical outcome [120].

Several studies have examined the utility of IL-8 as a diagnostic or prognostic marker in patients with ovarian cancer [121–123]. For example, increased IL-8 expression in ovarian cyst fluid, ascites, serum, and tumor tissue from ovarian cancer patients is found to be associated with high-grade and advanced-stage cancers, as well as with decreased disease-related patient survival [121, 123]. Collectively, these data provide the rationale for targeting IL-8 as a therapeutic approach in ovarian carcinoma. 

Decrease in IL-8 expression, especially when combined with taxane-based chemotherapy, led to a statistically significant reduction in orthotopic tumor growth. Xu and Fidler [36] reported that IL-8 overexpression was directly associated with increased tumor vascularity and tumor cell proliferation in ovarian carcinoma. 

#### 7.2.2. Enhancement of Cancer Mechanism in Melanoma

In melanoma, increased IL-8 levels are associated with increased tumor angiogenesis; conversely, a reduction in tumor microvessel density occurred following treatment with an anti-IL-8 antibody [124]. IL-8 has also been shown to increase tumor cell proliferation and to prolong the survival of human endothelial cells and enhance their ability to form tubules, which supports the theory that the proangiogenic effects of IL-8 are due to activation of both tumor and endothelial cells [113, 124].

Members of the MMP family of proteins promote tumor angiogenesis as well as cellular detachment, invasion, and metastasis, and some MMP family members, including MMP-2 and MMP-9, are reported to be regulated by IL-8 expression [125, 126]. IL-8 induces MMP-2 and MMP-9 expression in bladder cancer and melanoma cell lines, which contributed to increased tumor cell invasion *in vitro* [113, 126].

#### 7.2.3. Increase of VEGF and Neuropilin Expression in Pancreatic Cancer

IL-8 is upregulated in both cancer and chronic inflammatory diseases of the pancreas [127]. It is linked to pancreatic cancer tumorigenesis primarily through its regulation of angiogenesis and metastasis [120]. Human umbilical vein endothelial cells (HUVECs) proliferation and angiogenesis are both increased when cocultured with pancreatic cancer cells, or with exogenous in IL-8. The increase of cell proliferation and angiogenesis of HUVEC can be blocked by IL-8-neutralizing antibodies [128].

IL-8 is associated with chronic diseases of the pancreas [127]. It is overexpressed in most human pancreatic cancer tissues [120]. Higher IL-8 levels in pancreatic cancer patient serum are associated with significant weight loss [129]. The expression levels of IL-8 appear to correlate with their tumorigenic and metastatic potential in an orthotopic xenograft model [130]. Furthermore, treatment with exogenous IL-8 increases the invasiveness of human pancreatic cancer cell, while blocking IL-8 inhibited the growth of another human pancreatic cancer cell [131]. Blocking IL-8 in pancreatic cancer cells decreased their growth and their ability to attach to endothelial cells, suggesting that IL-8 is an autocrine mitogenic factor important for metastasis [132].

The expression of IL-8 can be induced by many stimuli including lipopolysaccharide, phorbol 12-myristate 13-acetate (PMA), IL-1, and TNF. Several stress factors, such as hypoxia, acidosis, nitric oxide (NO), and cell density, also significantly influenced the production of IL-8 in human pancreatic cancer cells [133].

IL-8 is involved in cancer hypoxia pathway where its expression is regulated by hypoxia-inducible factor-1 (HIF-1), NF-*κ*B, and KRAS [128]. IL-8 overexpressed in pancreatic cancer increases MMP-2 activity and plays an important role in the invasiveness of human pancreatic cancer [130, 131, 134], and human pancreatic cancer is associated with increased expression of IL-8 [127].

IL-8 as a proantigenic cytokine that helps the spread of distant metastasis by neovascularization and promotes the survival of the tumor mass in general by maintaining a rich capillary network to accommodate the heavy nutrient requirements of this aggressive cancer. Blocking IL-8 and IL-8 receptor CXCR2 significantly inhibited angiogenesis [118, 135].

Both IL-8 and VEGF are important components in cellular response to hypoxia, a common event in cancer, including human melanoma, colon cancer, and pancreatic cancer [117]. IL-8 acts as a direct growth and survival factor on pancreatic cancer cells, and IL-8 as multifaceted regulator of gene expression can regulate multiple pathways including angiogenesis, metastasis, and response to hypoxia in pancreatic cancer [136].

#### 7.2.4. Expression in the Neuroendocrine and Nonneuroendocrine Compartments of Prostate Cancer

Moore et al. demonstrated that IL-8 is a positive regulator of tumor formation in severe combined immunodeficiency (SCID) in mice injected with the prostate cancer cell line PC-3 [137]. Patients with PC (prostate cancer) have high serum levels of IL-8 which correlates with the stage of the disease. Additionally, in PC serum IL-8 levels have been determined to be an independent prognostic variable from the serum levels of free and total prostate-specific antigen (PSA) [138]. The combined use of free and total PSA ratio and IL-8 levels has been found to be more accurate in distinguishing between prostate cancer and benign prostatic hypertrophy.

In PC, serum IL-8 levels increase with progression of the disease [139]. The PC cell line 3 expresses and secretes IL-8 [140] and expresses IL-8 receptors CXCR1 and CXCR2 [140]. IL-8 is a mitogenic [21] and angiogenic factor [141]. PC cell line LNCaP does not express IL-8, but selection of the cells in androgen-deprived media led to the emergence of a cell line that produces IL-8 and is more tumorigenic than the parental cells [141].

The IL-8 receptor CXCR1 is rarely expressed in benign epithelial cells, its expression is increased in PIN (pancreatic invasive neoplasm), and further increase in invasive tumor suggests paracrine mechanism where IL-8 produced by the NE tumor cells may promote the proliferation of the non-NE tumor cells in the absence of androgen [142]. 

#### 7.2.5. Metastatic Factor in Breast Cancer

Estrogen receptor (ER) status is an important parameter in breast cancer management as ER-positive breast cancers have a better prognosis than ER-negative tumors. IL-8 is overexpressed in most ER-negative breast, ovary cell lines, and breast cancer, whereas no significant IL-8 levels are found in ER-positive breast or ovarian cell lines. IL-8 is considered as a potential metastatic factor in breast cancers [38]. IL-8 is found not only in normal but also in cancerous breast [37, 143].

Metastasis represents the major remaining cause of mortality in human breast cancer, which suggests that invasiveness is associated with lack of ER and changes in IL-8 expression. However, there was no correlation between ER*β* expression and IL-8 level, indicating ER*α*, the main estrogen receptor in ER-positive breast and ovarian cancer cells, is the receptor linked to IL-8 expression. Patients with recurrent prostate, breast, or ovarian cancer exhibit higher levels of IL-8 in serum or peripheral blood leukocytes [138, 144, 145] and in cancer tissues [34]. Several studies show that IL-8 expression in breast tumors is identical between normal and cancer tissue [146, 147].

Concerning IL-8 receptors, CXCR1 expression is extremely low in all lines, whereas most of the cells show a nice expression of CXCR2, without any correlation with ER status [143]. CXCR1 and CXCR2 which are encoded by two distinct genes [148, 149] are expressed in most cancer cells with no apparent correlation with the grade of the tumor [150, 151].

Exogenous expression of IL-8 increases by twofold the invasion rate of ER-positive breast cancer cells, without affecting the *in vitro *proliferation rate of these cells which is the proinvasive role of IL-8. When IL-8 is transfected in cancer cells, both tumor inhibition [152, 153] and promotion [126, 154] have been observed *in vivo *depending on the cell type [155].

#### 7.2.6. BEV (Epstein Barr Virus) and NPC (Nasopharyngeal Tumor)

Several chemokines secreted from EBV-infected NPC cells are increased upon EBV reactivation into the lytic cycle, and IL-8 is upregulated most significantly [156].

The most frequent histological type of NPC is closely associated with Epstein-Barr virus (EBV) infection [157]. NPC exhibits several inflammatory features in the tumor tissues, including intensive leukocyte infiltration, abundant expression of inflammatory cytokines, and constitutive activation of inflammation-associated transcription factors [158]. Expression of several chemokines has been demonstrated in NPC tumors, including IL-8, macrophage inflammatory proteins (MIPs), macrophage chemoattractant proteins (MCPs), and RANTES [159].

EBV reactivation in NPC cells is associated with the induction of certain chemokines where IL-8 was upregulated most significantly and consistently [147].

Neutrophils first invoke into inflamed tissues, then they produce a variety of chemokines with potentials to direct sequential recruitment of other leukocytes [160]. Therefore, by initial recruitment of neutrophils, IL-8 may trigger the subsequent influx of leukocytes in NPC. Notably, neutrophil infiltration promoted by tumor-derived IL-8 has been linked to the poor prognosis of bronchioloalveolar carcinoma and to increased genetic instability of Mutatect tumors [161], suggesting that IL-8-attracted neutrophils may contribute to tumorigenesis.

IL-8 is associated with the level of vascularization in NPC [159, 162]. Moreover, NPC is a highly metastatic cancer, and IL-8 may be involved in the phenotype since it can promote tumor invasion or metastasis through induction of certain metalloproteinases [154].

IL-8 is a converged target gene of gammaherpesviruses in both latent and lytic infection states. EBV utilizes the lytic protein Zta and the latent protein LMP1 to induce IL-8 expression, while Kaposi's sarcoma-associated herpesvirus (KSHV) can upregulate IL-8 by either the lytic protein K15 or the latent protein K13 [163]. Since KSHV-associated Kaposi's sarcoma also exhibits several inflammation-like features, induction of IL-8 is likely to be critical for the virus-mediated “inflammatory tumorigenesis”; [159, 162].

Blockage of IL-8 or IL-8 receptors may be considered a potential therapeutic approach for treating NPC or other inflammation-related malignancies [156].

## 8. Conclusion

IL-8, a potent angiogenic, proinflammatory, growth-promoting factor, properties which may be shared by other chemokines [164], is also a chemoattractant for neutrophils and induces expression of several cell adhesion molecules [164]. It also lead to neutrophil activation [165] and hence might contribute to the pathogenesis of inflammatory diseases. IL-8 specifically chemoattracts several cell types, which is the basis for inflammation. Neovascularization is a crucial step in tumor growth and metastasis. Regulation of IL8 production is a key mediator of inflammation by NF-**κ**B. The receptors for IL-8 are widely expressed on normal and various tumor cells.

IL-8 induces proinflammatory, chemotactic, and matrix, degradative responses in many pathologies. More research will certainly help to achieve a much better understanding of the function of IL-8 in different pathologies. However, knowledge gained from IL-8 data might be applied in a foreseeable future to cure the low back pain that often accompanies disc degeneration and therefore be beneficial for the patient. Despite exciting advances on IL-8, significant technical obstacles still have to be overcome before such approaches become realistic alternative therapeutic options to conventional surgical intervention procedures. Studies to understand IL-8 gene expression in the various cell types may lead to new therapeutics to enhance or inhibit IL-8 production. Many outstanding questions regarding IL-8 and inflammation exist. Further examination pin pointing the role of different IL-8 expressing subsets will allow us to better understand this cytokine.

## Abbreviations

IL: Interleukin
ROS: Reactive oxygen species
TNF-*α*: Tumor necrosis factor-*α*
TGF: Tumor growth factor
INF: Interferons
NGF: Nerve growth factor
BAL: Bronchoalveolar lavage
NF-*κ*B: Nuclear factor-*κ*B
MMP: Matrix metalloproteinases
VEGFR: Vascular endothelial growth factor receptor
GRO: Melanoma growth stimulatory activity
MIP: Macrophage inflammatory protein
NAP: Neutrophil-activating protein
CTAP: Connective tissue-activating protein
BK: Bradykinin
NGF: Nerve growth factor
BAL: Bronchoalveolar lavage
CF: Cystic fibrosis
PGE: Prostaglandin
AP: Activator protein
RSV: Respiratory syncytial virus
NP: Nucleus pulposus
HUVECs: Human umbilical vein endothelial cells
NO: Nitric oxide
HIF: Hypoxia-inducible factor
ER: Estrogen receptor
BEV: Epstein Barr virus
NPC: Nasopharyngeal tumor
MIP: Macrophage inflammatory proteins
MCP: Macrophage chemoattractant proteins
KSHV: Kaposi's sarcoma-associated herpesvirus.
